# Dr. Stephen G. Stevens

**Published:** 1904-11

**Authors:** R. R. Andrews

**Affiliations:** Cambridge


### DR. STEPHEN G. STEVENS.

I WRITE to notify you of the death of Dr. Stephen G. Stevens, of Boston. He was born in Brooks, Maine, in 1844. He served in the Civil War as a member of Company D, New York Frontier Cavalry; commenced the practice of dentistry in Lynn, Mass.; graduated at the Boston Dental College in 1877; bought the practice of the late Dr. R. L. Robbins and removed to Boston, where he has practised dentistry at 2 Commonwealth Avenue. Dr. Stevens was an honored member of the dental profession and was a very skilful operator. He was a trustee of the Boston Dental College; was ex-president of most of the dental societies in his city and State, and at the time of his death was a member of the Boston Society of Dental Improvement, the American Academy of Dental Science, the Northwestern Dental Society, and the Alumni Association of the Boston and Tufts Dental College. I thought perhaps you would wish to notice Dr. Stevens's death in the JOURNAL.

R. R. ANDREWS.

CAMBRIDGE, September 22, 1904.

